# The Impact of Cross-Cultural Adaptation on Entrepreneurial Psychological Factors and Innovation Ability for New Entrepreneurs

**DOI:** 10.3389/fpsyg.2021.724544

**Published:** 2021-10-28

**Authors:** Qianfu Zhou

**Affiliations:** School of Management, Wuhan University of Technology, Wuhan, China

**Keywords:** new entrepreneur, cross-cultural adaptation, entrepreneurial intention, individual psychological adaptation, entrepreneurial psychology

## Abstract

In the context of economic slowdown and unemployment upsurge in China, many college graduates set their goals on entrepreneurship. To help more new entrepreneurs improve their innovation ability and entrepreneurial success rate, the synergy theory of entrepreneurial psychology and entrepreneurial ability in the entrepreneurial period was put forward from the perspective of cross-cultural adaptation. On the basis of positive psychology, the entrepreneur’s psychological quality and innovation ability were combined to help explore the entrepreneur’s inner psychology and innovation ability. The study first analyzed the current development status and characteristics of new entrepreneurs through the literature review method, and explored the relationship between cross-cultural adaptation and new ventures. Second, it examined the relationship among achievement incentives, knowledge level, and the role of communication and entrepreneurs’ entrepreneurial intentions from the perspective of positive psychology. Finally, based on the influence relationship and personal experience, research hypotheses and models, the study verified the hypotheses by designing questionnaire surveys, and used the controlled variable method to conduct single-factor test analysis on influencing factors and perform a *t-*test on the data. The results show that the Cronbach’s α coefficient in the QS is 0.894, which has good reliability. According to the regression analysis results, achievement motivation (AM) can significantly affect entrepreneurial intention (EI) (β = 487, *p* < 0.05), knowledge level (KL) and EI (β = 316, *p* < 0.05), and the regression coefficient is greater than 0, but there is no significant effect of communicate effectiveness (CE) on EI (*p* = 0.109 > 0.05). In the control variables influence analysis, the level of education of new entrepreneurs was negatively correlated with AM. When the college students’ educational level improved, their EI decreased. It shows that AM can promote new entrepreneurs to pursue higher goals, and different level of education has a greater impact on entrepreneurs’ AM, which has a more significant impact on entrepreneurial intention. The results further prove that entrepreneurs’ psychological factors have a particular impact on the development of enterprises.

## Introduction

Since the twentieth century, the world is gradually entering a new era—the era of knowledge economy. It mainly relies on high-tech and innovative talents to promote the sustained and rapid development of the social economy, with the development of science and technology as the driving force. Entrepreneurship economy is an important part of the knowledge economy. Entrepreneurship plays an increasingly important role in promoting economic development. Therefore, it can be said that the era of knowledge economy is the era of entrepreneurship. New entrepreneurs are the main force in the era of knowledge economy. Their entrepreneurial development always affects the trend of social economy. In order to improve the innovative ability and entrepreneurial psychological capital of new entrepreneurs, it is necessary to conduct systematic research on them (Díaz-García and Jiménez-Moreno, 2010; Catherine et al., 2020; Cai et al., 2021). Entrepreneurial competence involves adaptive behaviors and strategies, which can influence the behaviors of related personnel and promote entrepreneurs’ innovation ability. Besides, the innovation ability determines the core competitiveness and future development potential of an enterprise. Hence, enterprises should acquire and maintain continuous innovation abilities to remain competitive. Along with China’s vigorous promotion of the development of entrepreneurship education, entrepreneurship education has also entered a period of rapid development, and research related to entrepreneurship education is constantly deepening (Souitaris et al., 2007; Sirbu et al., 2015; Anggadwita et al., 2021; Leung et al., 2021).

Here, based on the theory of positive psychology, the hypotheses were constructed for the relationship between entrepreneurial psychology and the positive psychology of new entrepreneurs. Then, the QS (Questionnaire Survey) was designed and distributed to the new entrepreneurs who have just graduated to verify the hypotheses. Subsequently, the statistical results were analyzed through multiple linear regression and discussed and compared with previous studies. Meanwhile, the significance and future research direction were explored to reflect the impact of cross-cultural adaptation on the innovation ability and psychological state of entrepreneurs in China. Finally, based on the survey results, enlightenment and suggestions were advanced for relevant practitioners and researchers (Zhang et al., 2014; Zheng et al., 2018; Wu Y. J. et al., 2020; Xie et al., 2020; Yin et al., 2020; Yuan and Wu, 2020).

In order to help new entrepreneurs adapt to the cross-cultural development environment more quickly and improve their entrepreneurial psychological quality, this article, based on the existing research results on the psychology of new entrepreneurs and cross-cultural adaptation, provides an analysis of the entrepreneurial psychological quality of new entrepreneurs. The meaning and content were interpreted in detail, the psychological quality problems in the entrepreneurship process were sorted out and analyzed by combining with the questionnaire, the causes were examined, and an effective method of entrepreneurial psychological quality and innovative ability were proposed on the basis of the relevant knowledge and theories of positive psychology (Wu and Chen, 2021).

The research value lies in that by studying the relationship and influence mechanism between the psychological capital and cultural adaptation of new entrepreneurs, the specific role of psychological capital in the entrepreneurial process can be verified, thus further analyzing the influencing factors in the entrepreneurial process and helping entrepreneurs get achievements. The study intends to develop a psychological capital development model for the entire group of entrepreneurs on the basis of other scholars’ research on psychological capital development, reflecting the practical orientation of this research. The innovation includes the combination of cross-cultural adaptation with the entrepreneurial psychology of new entrepreneurs, and the study on the innovative ability and psychological level of new entrepreneurs from the perspective of positive psychology (Masgoret and Ward, 2006; Misoska et al., 2016; McClelland et al., 2018; Moses and Kisubi, 2021).

The contribution of the proposal is reflected in three aspects. Firstly, the cross-cultural adaptation of new entrepreneurs is explored from the perspective of cultural psychology, which fills a vacancy in the academic field. The research results of EI and psychological factors have great influence in Chinese academic circles, but few scholars pay attention to the research of cross-cultural adaptation of new entrepreneurs. Secondly, based on abundant sampling data, various analysis methods are adopted for in-depth research and a more convincing conclusion, thereby overcoming the common problems in the research of EI assessment in China, such as insufficient sample size and lack of persuasion. Thirdly, some suggestions are advanced to help the new entrepreneurs overcome the influence of cross-cultural adaptation to the entrepreneurial environment, thus encourage their EI and innovation ability in an adverse environment (Qian et al., 2018; Rogoza et al., 2018; Mao and Ye, 2021; Neslihan et al., 2021; Otache et al., 2021; Pérez et al., 2021; Racine, 2021).

## Theoretical Basis and Hypothesis

### Research Status

In the early days, many scholars in the United States had the following views on entrepreneurship. First, Gartner believed that the personality traits and entrepreneurial results of entrepreneurs ultimately determined the essence of entrepreneurship; entrepreneur’s character and entrepreneurial results determined the essence of entrepreneurship. Second, Jeffrey Dimons, “Father of American Entrepreneurship Education,” held that entrepreneurship education was not only the integration of multiple forms and stages of organizations and enterprises, but should help entrepreneurs achieve their life goals and realize their life values. Important channels: Empirical evidence shows that the success of foreigners’ entrepreneurship can greatly affect the culture of the host country (Deng et al., 2021; Leung et al., 2021). At the same time, new entrepreneurs who receive diversified cultural education will inevitably compare the gap between the external environment and the adaptability in the entrepreneurial process. Intercultural competence refers to the ability of an individual to live and work effectively under different cultural backgrounds. This requires foreign employees without relevant cultural backgrounds to think and behave like people in the host country (Chen, 2019). It is worth noting that prospective entrepreneurs have four characteristics: trust, profit, learning and social interaction, of which trust is the most important (Wu and Song, 2019). Previous studies have shown that in the process of cross-cultural adaptation, investment in cross-cultural training by multinational companies has improved the overall quality of foreigners (Contreras et al., 2021). In China, many college students preparing to start a business are often caught in a dilemma, because school education is separated from the social entrepreneurial environment, which greatly affects their EI (Entreprenurial Intention). With their unique level of education, when they enter a new cultural environment and interact with strangers, their behavior and thoughts will inevitably be affected by cult neurogenes. For example, the occurrence of culture shock is due to differences in language, behavior, social means, and values, which affect the mental state of new entrepreneurs and lead to obvious EI (Ward, 2001). Therefore, the enterprise’s psychological adaptability is insufficient, and it is difficult to exert the innovation ability.

### The Entrepreneur’s Psychological Capital

Psychological capital exerts its effects in the form of three variables: The first form is an independent variable, which reflects the influence of psychological capital on the outcome variable; the second form is an intermediary variable, which reflects the influence of psychological capital on the independent variable and outcome. The mediating effect between variables; The third form is a moderating variable, which reflects the moderating effect of psychological capital on the relationship between two variables. Scholars’ cognition of entrepreneurial psychological factors is limited to entrepreneur’s personality traits (such as autonomy, control points, achievement needs and tolerance of ambiguity), and they have not been able to find states or state-like psychology that are very different from idiosyncratic psychological factors. With the rise of social cognition theory, especially positive psychology and positive organizational behavior, the role of entrepreneurial psychology factors in the model of new venture performance determining factors will be re-recognized and positioned (Llewellyn and Wilson, 2013; Gudykunst, 2020; Hande and Faruk, 2021; Serrano et al., 2021; Su et al., 2021; van Ewijk and Weber, 2021).

### The Relationship Between Psychological Capital

The self-interest motivation of entrepreneurs can be divided into material motivation and AM. The research of achievement needs proves the psychological feature difference between entrepreneurs and non-entrepreneurs in innovation and AM. Entrepreneurs need more achievement than others (Zeffane, 2013; Feng and Chen, 2020). AM and personal goals are the unmet needs of entrepreneurs, which are usually reflected through entrepreneurial behavior (Lam et al., 2017).

Researchers believe that people with higher education are more likely to engage in dynamic innovation activities than people with lower education. These innovation activities need scientific planning and require individuals to take responsibility for the outcome. Meanwhile, senior talents should also focus on tasks that involve skills and efforts, provide clear performance feedback, and are moderately challenging or risky. Particularly, entrepreneurial positions have a higher demand for these characteristics. However, university achievement cannot fully exert new entrepreneurs’ AM (Gomes et al., 2021). Therefore, new entrepreneurs should master specific knowledge and skills for organizing teams to deal with risks.

**FIGURE 1:**
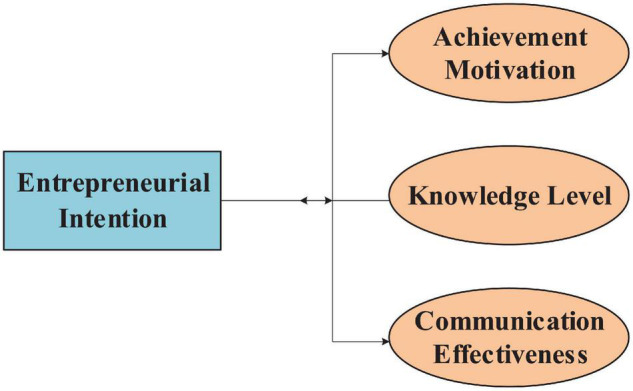
The relationship between EI and AM, KL, CE.

The existing cross-cultural adaptation situation shows that interpersonal communication is crucial to cross-cultural adaptation. Individuals who can effectively communicate with the locals can better acquire survival skills and cope with stress, thus exhibiting a higher level of psychological adaptation. Assistance to ex-pats and their families can help them better adapt to the host country can improve their living standard (Thompson, 2009). Therefore, besides mastering knowledge, and skills, and AM, the college new entrepreneur should also seek partners from external companies to confront challenges from the strange cultural environment (Guo and Hu, 2014). The impact of CE is divided into the following four areas. The structure of the relationship between EI and AM, KL and CE is shown in Figure 1:

1. Communication between work and local people: CE is affected by the character and work attitude of the local people.
2. Communication between work and team: CE is influenced by organizational model, management model, and job satisfaction.
3. Communication between work and family: CE is affected by daily life and working time.

Based on the results of previous research and ideas, the following hypotheses are put forward.

H1:AM has a positive impact on the EI of new entrepreneurs.

H2:KL has a positive impact on the EI of new entrepreneurs.

H3:CE has a positive impact on the EI of new entrepreneurs.

### Methods

#### Samples and Subjects

The research subjects are the entrepreneurial college students in China who have received western higher education. Through communication with the relevant business leaders, the QS was distributed to the new entrepreneurs who settled in the business incubator on the campus. A total of 400 QSs were distributed, 379 QSs were recovered, 12 invalid QSs were deleted due to blank and unverified answers, and 367 valid QSs were finally obtained, with an effective rate of 91.75%. To prevent extreme errors, the stratified sampling was used. All of the scale items had been modified to suit the Chinese cultural background and were based on a five-point Likert scale, with 1 point indicating “strongly disagree” and 5 points indicating “strongly agree.”

Research Methods: In this study, model fitting and six index tests were used, and the six indices consist of χ^2^/DF (Chi-square of Degrees of Freedom), GFI (Goodness of Fit Index), AGFI (Adjusted Goodness of Fit Index), NFI (Normalized Fit Index), CFI (Comparative Fit Index), and RMSEA (Root Mean Square Error of Approximation).

Their academic level was scored with a 4-point system, in which 1 point represents “sophomores,” and 4 points stand for “postgraduates.” The entrepreneurship education scale was designed based on students’ participation in innovation and entrepreneurship education and included three sub-scales, with a total of 10 questions. Entrepreneurship education provided students with the necessary information, knowledge, and other resources, thus forming a strong atmosphere of innovation and entrepreneurship, reducing environmental uncertainty, and creating a good environment for innovation and development. Here, several of these questions were selected.

Previous studies have explored the impact of education on EI. Entrepreneurship education is considered to be one key factor to improve EI. Many researchers have confirmed that there is a positive correlation between entrepreneurship education and EI.

Control Variables: Here, control variables include gender, level of education, technology, and other objective factors of entrepreneurs. Part of the QS design is shown in Table 1.

**TABLE 1:** Part of the QS.

**AM**	**AM**
01	I will take initiative to solve problems in my work.
02	I am concerned about my job responsibility
03	I will update professional skill when working as an entrepreneur.
04	At work, I show great interest.
KL	KL
01	I have participated in entrepreneurship and innovation skill training.
02	I think I know the policy information on entrepreneurship and innovation.
03	I have a lot of knowledge about management.
CE	CE
01	When communicating with people from different cultural backgrounds, I will change my position.
02	When communicating with people from different cultural backgrounds, I will express my idea clearly.
03	When communicating with people from different cultural backgrounds, I will provide feedback on the conversation.
EI	EI
01	I think I will start my business soon.
02	I have considered running my own business.
03	If given a chance to make a free decision, I will choose to start my own business.
04	Consider the current situation and various restrictions (such as capital), I will still choose to start my own business.

### Analysis of Research Results

The reliability and validity of the QS were tested through the SPSS 22.0 and AMOS 22.0, and the proposed hypotheses were verified using the structural equation model.

Data Collection: Among the investigated subjects, 65.4% were males, and the obtained household income distribution was basically in line with China’s household income statistics. Meanwhile, 68.4% of the respondents majored in management and economics, 23.2% of them majored in science and engineering, 5.2% of them majored in pedagogy, and 3.0% of them majored in pharmacy and medicine. Each item was scored with a 5-point Likert scale, from 1 point (strongly disagree) to 5 (strongly agree). It was shown in Table 2.

**TABLE 2:** Basic information of students.

**Variable**	**Items**	**Frequency**	**Percentage (%)**
Gender	Male	240	65.4
	Female	127	34.6
Household income (CNY/year)	Below 20k	12	3.3
	20k–50k	24	6.5
	50k–100k	65	17.7
	100k–500k	180	49.0
	500k–2,000k	82	22.1
	Over 2,000k	5	1.4
Academic discipline	Economic management	251	68.4
	Science and engineering	86	23.2
	Pedagogy	19	5.2
	Pharmacy and medicine	11	3.0
Level of education	Sophomore	54	14.7
	Junior	81	22.1
	Undergraduate	182	49.6
	Postgraduate	50	13.6

### Reliability and Validity Test

Here, the reliability of the QS of the new entrepreneurs was tested using the Cronbach’s α coefficient: if its value is greater than 0.7, the reliability is good. The results of a small sample survey showed that Cronbach’s α coefficient is 0.894, so the QS has good reliability.

Afterward, samples were analyzed through KMO (Kaiser Meyer Olkin) test. The results showed that the KMO is 0.903, the Chi-square is 2171.304, the significant probability is significant at the level of 0.05, and the cumulative variance contribution of these factors is 61.127%. Thus, the sample data are relevant and suitable for factor analysis.

**FIGURE 2:**
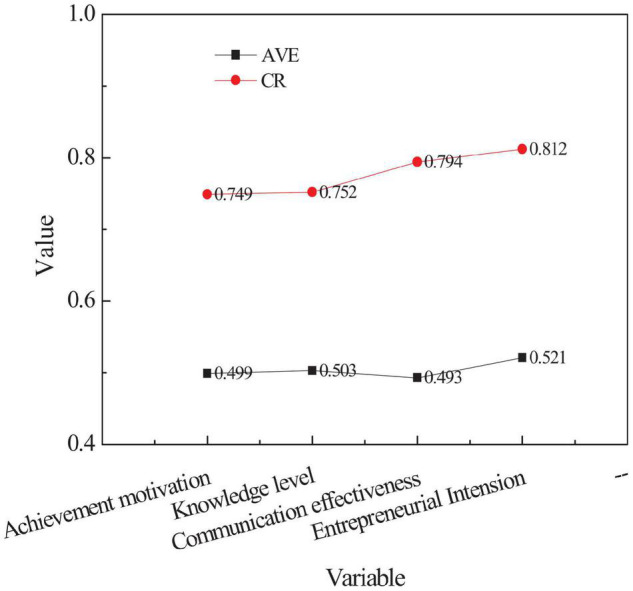
Comparison of AVE and CR mean-variance of Variables.

Figure 2 and Table 3 reveal that the confirmatory factor analysis on entrepreneurial psychology can be obtained, and the average variance extraction of the communication level and the comprehensive reliability score are the highest.

**TABLE 3:** Variable factor load.

**Variable**	**Item**	**Estimate**	**SE**
AM	KL1	0.769	
	KL2	0.661	0.083
	KL3	0.685	0.077
KL	CE1	0.67	
	CE2	0.771	0.113
	CE3	0.684	0.105
CE	EI1	0.705	
	EI2	0.597	0.072
	EI3	0.719	0.09
	EI4	0.777	0.082
EI	AM1	0.778	
	AM2	0.594	0.074
	AM3	0.755	0.07
	AM4	0.746	0.07

The reliability, convergent validity, and discriminant validity are analyzed using CFA (Confirmatory Factor Analysis) for the test variables. The results of CFA are shown in Table 3. The AVE (Average Variance Extraction) of each variable is greater than or equal to 0.5, indicating that the scale has satisfactory convergence validity. The CR (Comprehensive Reliability) values are greater than 0.7, indicating that the scale has satisfactory reliability.

### Model Fit

Table 4 lists the actual values of the model fitting indices, as well as their recommended and acceptable values. Apparently, all the main indices are within the acceptable range, which indicates that the constructed theoretical model well fits the sample data.

**TABLE 4:** Overall fitting index.

**χ^2^/DF**	**RMSEA**	**GFI**	**AGFI**	**IFI**	**NFI**	**CFI**
2.891	0.072	0.927	0.891	0.938	0.908	0.937
≤3.0	≤0.1	≥0.9	≥0.85	≥0.9	≥0.9	≥0.9

In demographic variables, research reveals that KL is positively correlated with the length of education of new entrepreneurs. The longer the education of a new entrepreneur is, the higher the level of knowledge is. Single-factor ANOVA (Analysis of Variance) shows that new entrepreneurs with longer years of education score higher in KL (*p* = 0.91 > 0.05), which is consistent with actual situations. Additionally, another result shows that there is no statistically significant difference in the three dimensions of new entrepreneurs engaged in different majors, it was shown in Table 5.

**TABLE 5:** The differences of new entrepreneurs.

		**In the KL dimension**				
		**Sum of squares**	**Degree of freedom**	**Mean square**	***F***	**Significance**
KL	Inter-group	7.613	5	1.523	1.913	0.091
	Intra-group	287.303	361	0.796		
	Total	294.916	366			

The estimated correlation between the measurement factors is calculated with the Pearson correlation coefficient. The variance is set to 1 (that is, the data are set to the normalized coefficients; Pearson correlation), and factor correlation coefficients are calculated using the estimation program. Table 6 displays that there is a significant relationship between the three dimensions of CE (self-awareness, social awareness, and feedback ability), the four dimensions of AM (problem-solving, sense of responsibility, learning motivation, and work interest), and the four dimensions of EI.

**TABLE 6:** Correlation coefficients of potential variables.

	**AM**	**KL**	**CE**	**EI**	**Total**
AM	1				
KL	0.515**	1			
CE	0.417**	0.486**	1		
EI	0.672**	0.604**	0.424**	1	
Total	0.814**	0.821**	0.717**	0.847**	1

**p < 0.05.

The linear regression fit is good, *R*^2^ = 0.545, which well reflects the impact of AM, KL, and EC on new entrepreneur’s EI, it was shown in Table 7.

**TABLE 7:** Coefficient statistics.

		**Unstandardized**		**Standardized**		**Collinearity**	
		**coefficient**		**coefficient**		**statistics**	
**Model**		**B**	**Standard error**	**Beta**	**T**	**Tolerance**	**VIF**
	(Constant)	0.429	0.167		2.573		
	AM	0.487	0.043	0.476	11.240	0.698	1.432
	KL	0.316	0.043	0.326	7.408	0.646	1.548
	CE	0.071	0.044	0.067	1.608	0.726	1.377

All the VIF (Variance Expansion Factor) is less than 5, which implies that the operation result is true and reliable, and the object does not have multicollinearity. The regression equation is significant, *F* = 145.118, and *p* < 0.01, which indicates that linear regression is accurate and reliable to investigate the influence of dependent variables KL and AM on EI, and at least one independent variable can significantly affect EI. Meanwhile, AM can significantly affect EI (β = 487, *p* < 0.05), KL can also significantly affect EI (β = 316, *p* < 0.05), and the regression coefficients are greater than 0. While CE has no significant effect on EI (*p* = 0.109 > 0.05). Finally, the regression equation between the variables is as follows.

EI = 0.167 + 0.487*AM + 0.316*KL

Hence, hypothesis 1 and hypothesis 2 are verified, while hypothesis 3 is not verified.

## Results

**FIGURE 3:**
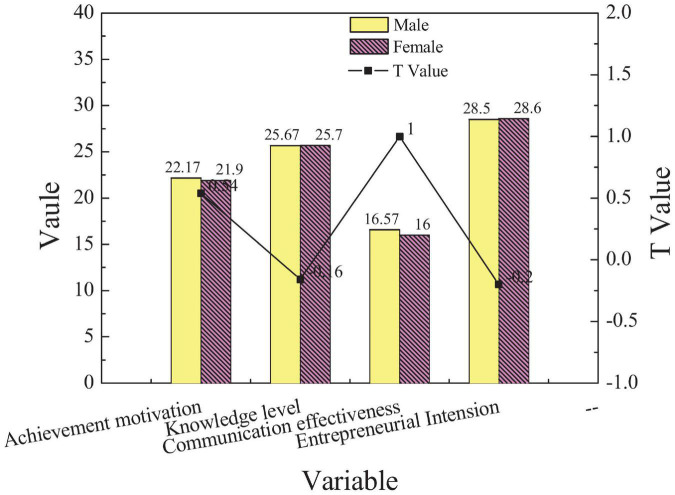
Comparison of scores of new entrepreneurs of different genders in different EI dimensions.

Firstly, entrepreneurs of different ages were evaluated through the independent sample *t*-test analysis on different dimensions. The results are shown in Figure 3.

**FIGURE 4:**
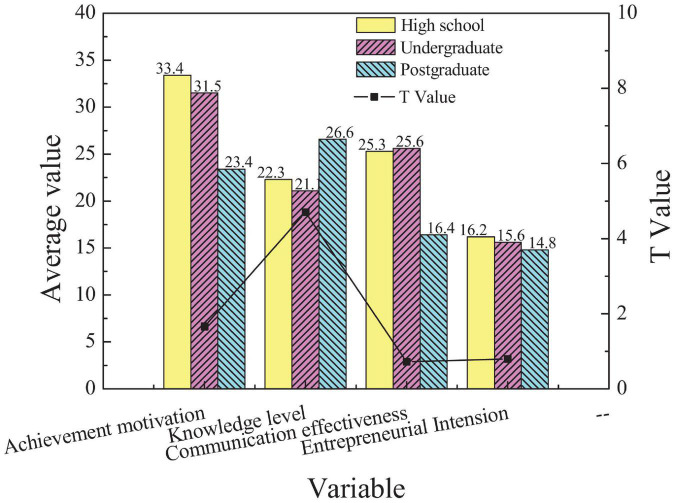
Comparison of scores of entrepreneurs with different level of education in different EI dimensions.

Figure 3 shows that the scores of male and female entrepreneurs are lower than 3 in all dimensions. The scores of male entrepreneurs are slightly higher than those of female entrepreneurs. Especially in terms of entrepreneurial cognition, male entrepreneurs are higher than female entrepreneurs. The difference in entrepreneurial psychology is not obvious (*p* < 0.05), which shows that gender is not the main factor affecting entrepreneurs’ entrepreneurial psychology. Secondly, entrepreneurs with different levels of education were evaluated through the independent sample *t*-test analysis on different dimensions, and the results are shown in Figure 4.

Figure 4 suggests that entrepreneurs with different levels of education have the most significant difference in dimension AM (*p* < 0.05). Specifically, senior high school degree has the highest AM, while postgraduate has the lowest AM, which indicates that there is a negative correlation between the level of education and AM. The possible reason is that the higher the level of education is, the stronger the cognitive ability is, and the clearer the cognition of their shortcoming is. Besides, with a higher level of education, students have a more comprehensive understanding of social pressure and entrepreneurial risks, so the AM becomes lower.

**FIGURE 5:**
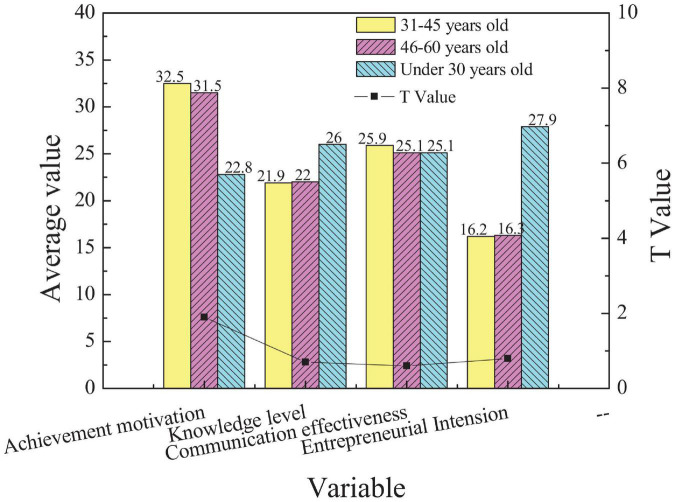
Comparison of scores of new entrepreneurs of different ages in different EI dimensions.

Thirdly, The entrepreneurs of different ages were evaluated using the independent sample *t*-test analysis on different dimensions. The results are shown in Figure 5.

Figure 5 demonstrates that there is no significant difference in the four dimensions of EI among entrepreneurs of different ages (*p* > 0.05), indicating that age has no obvious effect on EI.

According to the above research results, gender and age are not the main factors affecting the entrepreneurial psychology of new entrepreneurs, but different educational backgrounds are the main factors affecting entrepreneurs’ entrepreneurial psychology, and people with high school education have the highest entrepreneurial intentions. Graduate students People above and above have the lowest entrepreneurial intentions, and there are significant differences in the effectiveness of entrepreneurial psychological capital at different levels. The performance of new entrepreneurs with a high level of education is higher than that of entrepreneurs with a secondary level of psychological capital. Enterprise performance, and the latter’s new venture performance is higher than the new venture performance of entrepreneurs with low educational level psychological capital.

## Conclusion

In order to help new entrepreneurs adapt to the cross-cultural development environment more quickly and improve their entrepreneurial psychology, the study takes the entrepreneurial psychology of new entrepreneurs as the research object, based on the existing new entrepreneurs’ psychology and cross-cultural adaptation. A detailed interpretation was made of the meaning and content of the entrepreneurial psychology of new entrepreneurs, a single-factor descriptive statistical analysis was conducted on entrepreneurs’ entrepreneurial psychology through questionnaire and confirmatory factor analysis. Based on the current findings, several conclusions can be drawn. The results show that the difference in entrepreneurial psychology between male entrepreneurs and female entrepreneurs is not obvious (*p* < 0.05), indicating that gender is not the main factor affecting entrepreneurs’ entrepreneurial psychology; while entrepreneurs with different educational levels have different levels of AM (*p* < 0.05), and the higher the level of education is, the lower the entrepreneurial motivation is; finally, entrepreneurs of different ages have no significant difference in the four dimensions of EI (*p* > 0.05), indicating that age has no significant effect on EI.

First, AM and KL have a positive impact on EI. The positive impact of CE on EI is not supported or only partially supported. Second, when the KL of college students is improved, their EI is reduced. However, by avoiding blind entrepreneurship, college students can better adapt to the cross-cultural environment. College education can increase the positive views of new entrepreneurs on entrepreneurship by cultivating management ability, publicizing entrepreneurship policies, and creating an entrepreneurial atmosphere. The AM can also motivate new entrepreneurs to engage in entrepreneurial activities. The results show that AM can promote new entrepreneurs to pursue higher goals, and different level of education has a greater impact on the entrepreneur’s AM, thereby significantly impacting EI.

## Limitations and Future Research

The samples of the survey are all from college students with similar cultural backgrounds in same country. Stratified sampling is used instead of random sampling, which may affect the representative nature of the experiment. Due to the requirements of cross-cultural adaptation, all the investigated new entrepreneurs are sampled from the top universities in China and have received systematic professional education. Some analysis shows that professional course training also affects students’ career choices, thus leading to different attitudes and intentions toward entrepreneurship. These environmental and cultural factors greatly increase the commonness of the special sample groups, resulting in non-sampling errors. Therefore, it is necessary to expand the cultural background richness of the sample and determine the causal relationship through experimental design in the future research (Van and Clelland, 2015; Wu and Wu, 2017; Fadzil et al., 2019; Wu W. et al., 2020).

Meanwhile, the longitudinal analysis will be considered to explore the gap in the subsequent entrepreneurial process of new entrepreneurs under the cross-cultural background due to EI difference, and the impact of these differences on entrepreneurial behavior and decision-making will be further confirmed.

## Data Availability Statement

The raw data supporting the conclusions of this article will be made available by the authors, without undue reservation.

## Ethics Statement

The studies involving human participants were reviewed and approved by Wuhan University of Technology Ethics Committee. The patients/participants provided their written informed consent to participate in this study. Written informed consent was obtained from the individual(s) for the publication of any potentially identifiable images or data included in this article.

## Author Contributions

The author confirms being the sole contributor of this work and has approved it for publication.

## Conflict of Interest

The author declares that the research was conducted in the absence of any commercial or financial relationships that could be construed as a potential conflict of interest.

## Publisher’s Note

All claims expressed in this article are solely those of the authors and do not necessarily represent those of their affiliated organizations, or those of the publisher, the editors and the reviewers. Any product that may be evaluated in this article, or claim that may be made by its manufacturer, is not guaranteed or endorsed by the publisher.
